# Reproductive responses of dairy cows with ovarian cysts to simultaneous human chorionic gonadotropin or gonadotropin-releasing hormone and cloprostenol compared to gonadotropin-releasing hormone alone treatment

**DOI:** 10.14202/vetworld.2015.640-644

**Published:** 2015-05-22

**Authors:** T. Taktaz, M. Kafi, Adel Mokhtari, M. Heidari

**Affiliations:** 1 Department of Animal Clinical Science, Shahrekord Branch, Islamic Azad University, Shahrekord, Iran; 2 Department of Animal Reproduction, Faculty of Veterinary Medicine, Shiraz University, Shiraz, Iran

**Keywords:** cloprostenol, dairy cows, GnRH, hCG, ovarian cyst

## Abstract

**Aim:**

Bovine ovarian cysts are a common cause of economic loss in modern dairy herds. The objective of the present study was to evaluate the reproductive responses to three protocols using hCG, GnRH and cloprostenol when the definite diagnosis of the type of ovarian cyst is/is not possible in dairy cows.

**Materials and Methods:**

A total of 144 lactating dairy cows with ovarian cysts were divided into three groups. At diagnosis (Day 0), cows in Group 1 (the conventional method, n=47) were injected with 0.02 mg of a GnRH analogue i.m. (Buserelin); cows in Group 2 (n=47) were intramuscularly treated with 0.02 mg Buserelin plus 500 µg cloprostenol; and cows in Group 3 (n=50) were intramuscularly treated with 1500 IU hCG plus 500 µg cloprostenol. All cows received 500 µg cloprostenol intramuscularly on Day 10.

**Results:**

No statistically significant differences were found in the recovery time, interval to conception, conception rate at first AI, and pregnancy rates by Days 70 and 100 after treatment among the three groups.

**Conclusions:**

Simultaneous treatment of ovarian cysts with hCG or GnRH and cloprostenol appeared to have no advantage over the conventional method, GnRH alone, in dairy cows. Furthermore, hCG and GnRH have an equal therapeutic effect in cows with ovarian cysts.

## Introduction

Ovarian cysts are defined as follicular structures with a diameter of at least 25 mm that fail to ovulate, and remain on one or both ovaries of the cow in the absence of any active luteal tissue for an extended period of time in which they interfere with normal ovarian cyclicity [1,2]. Ovarian cysts are one of the most important causes of infertility in dairy cows and are associated with considerable economic loss through their high incidence, increase in days to first service, and increase in days open [3,4]. The incidence of ovarian cysts in dairy cows ranges from 2.7% to 15.1% [5-8]. The exact cause of ovarian cysts is not presently known, but it appears that disruption of the hypothalamo-pituitary-gonadal axis, by endogenous and/or exogenous factors, causes cyst formation [1,9].

Different approaches including manual rupture [10], cystic fluid aspiration [11,12], and hormonal applications have been used in the treatment of ovarian cysts with the hormonal treatment being the most common approach [13-15]. Gonadotropin-releasing hormone (GnRH) and its analogues have frequently been used for the treatment of luteal or follicular ovarian cysts and proved to be successful [7,16-18]. Human chorionic gonadotropin (hCG) has also been demonstrated to be a successful treatment for ovarian cysts [19]. It was reported that hCG and GnRH have an equal therapeutic effect in cystic dairy cows [19,20]. Prostaglandin F_2α_ (PGF_2α_) or its analogues are the treatment of choice for luteal cysts [16,21] and has also been used as the initial treatment of bovine ovarian cysts [22].

Differentiation between follicular and luteal cysts by rectal palpation has not been very accurate [23,24]. Accuracy with ultrasonography is much greater but is not absolute [24]. The only accurate way to differentiate between luteal and follicular cysts is the use of on-farm progesterone kits in which their use has not been widely adapted in many countries. Therefore, a treatment protocol which works well for both luteal and follicular cysts may be more valuable than a specific treatment for each type of cyst. Because differentiation between luteal and follicular cysts is difficult, combining PGF_2α_ with a luteotrophic agent (GnRH or hCG) may be more beneficial compared to GnRH/hCG or PGF_2α_ alone at the time of cyst diagnosis. In this regard, it was shown that administration of a GnRH analogue (buserelin) simultaneously at the time of cloprostenol injection did not interrupt the luteolytic effect of cloprostenol on the normal corpus luteum or luteal cysts [25,26]. On the other hand, there is no evidence indicating the inhibitory effect of cloprostenol on the luteinizing effect of GnRH or its analogues. Contradicting information is available on the effectiveness of the simultaneous application of GnRH with PGF_2α_ at the commencement of the conventional ovarian cysts treatment in dairy cows [13,26,27].

Furthermore, there is no report about the simultaneous application of hCG with PGF_2α_ in the treatment of ovarian cysts. Our hypothesis was that simultaneous administration of hCG or GnRH with cloprostenol could treat the undiagnosed type of ovarian cysts similarly to the conventional treatment (using GnRH and 10 days later cloprostenol administration). Therefore, we designed the following experiment to compare the response of cows with ovarian cysts given simultaneous treatment with GnRH or hCG and cloprostenol, and cloprostenol alone 10 days later to the response of those treated with the conventional treatment in Holstein dairy cows.

## Materials and Methods

### Ethical approval

This study was approved by the Center of Excellence for Studying Reproduction in High Producing Dairy Cows, Shiraz University, Iran.

### Animals and study farm

The study was conducted from March to October 2013 on a commercial dairy farm, milking approximately 1500 Holstein cows, in Shahrekord, Iran. The cows were milked 3 times a day with a herd average annual milk yield of 9500 kg/cow. Cows were housed in free-stall barns and fed a total mixed ration. The voluntary waiting period was 45 days and 55 days for multiparous and primiparous cows, respectively. Body condition score was evaluated on a 5-point scale at parturition and at peak lactation (i.e., 50-60 days in milk [DIM]). Heat detection was done by visual observation three times a day. Cows were examined by transrectal palpation and ultrasonography via a 5 MHz linear-array transducer (CTS-900V, SIUI, Japan) during weekly herd visits. All ultrasound examinations were performed by the same clinician. Cows with rectal temperature higher than 39.5°C, abnormal postpartum uterine discharge, adhesions, pyometra, urovagina, and pneumovagina were not used in the study. Furthermore, cows with a history of clinical endometritis, mastitis, lameness, and digestive disorders in the current lactation, cows diagnosed to have clinical endometritis by transrectal palpation, and cows with echogenic fluid at uterine lumen in ultrasonographic examination (presumed to have clinical endometritis) were not used in the study.

The ovaries of each cow were then examined by transrectal ultrasonography. Cows with follicles >25 mm in diameter in the absence of luteal structure were considered to have ovarian cysts [1,2]. Cysts with a wall thickness of ≥3 mm and gray echogenic patches along the inner wall or within the antrum of the cyst were classified as luteal, and cysts with a wall thickness of <3 mm with homogenous anechoic antrum were classified as follicular cyst [1].

### Study design

Selected cows (n=144) were at 35-120 (48±1.7) DIM and had a body condition score between 2.5 and 3.5 on the day of cyst diagnosis. The parity of the cows ranged from 1 to 6. Cows included in the study had not already been treated for cysts in the current lactation. Cows were randomly allocated to one of the three treatment groups at the time of reproductive system examination in the manner that the total number of cows, the number of follicular cysts, and the number of luteal cysts in three groups be nearly similar. Treatment groups were not significantly different in the mean DIM (45.2, 48.6, and 50.2 for Groups 1, 2, and 3, respectively; p=0.75). At diagnosis (day 0), cows in Group 1 (n=47) were injected intramuscularly with 0.02 mg of a GnRH analogue (conventional treatment, Buserelin); cows in Group 2 (n=47) were simultaneously treated with 0.02 mg buserelin (i.m.) and 500 µg of a PGF_2α_ analogue (cloprostenol, i.m.); and cows in Group 3 (n=50) were simultaneously treated with 1500 IU hCG (corulon, i.m.) and 500 µg cloprostenol (i.m.). All cows received 500 µg cloprostenol (i.m.) on day 10 after GnRH or hCG administration. Cows which exhibit estrus during or after treatment were then artificially inseminated using the semen of high-fertility proved bull 12 h after estrus. The proposal of the present research was approved by the Committee of Scientific Research and Animal Ethics of School of Veterinary, the Islamic Azad University of Shahrekord.

### Pregnancy diagnosis

All cows were examined for pregnancy by transrectal ultrasonography via a 5 MHz linear array transducer (CTS-900V, SIUI, Japan) on day 35±3 after artificial insemination (AI). Conception rate was calculated as the proportion of inseminated cows pregnant at the time of pregnancy diagnosis.

### Recording reproductive indices

Recorded data included parity, recovery time (interval to first estrus), interval to conception, conception rate at first AI after treatment, and pregnancy rates by days 70 and 100 after treatment.

### Statistical analysis

Data were analyzed using SAS (version 9.2, SAS Inst. Inc., Cary, NC). The one-way ANOVA procedure was used to test the hypothesis that the means of the considered factor between the three treatment groups are not significantly different. Using the one-way ANOVA procedure, normality and homogeneity of variance were checked by Kolmogorov–Smirnov and Levene’s homogeneity-of-variance tests, respectively. The Kruskal–Wallis test was used to determine whether or not the values of a particular variable differ between the three treatment groups when the assumption of ANOVA (normality) was not met. The measures of association such as Pearson Chi-square and Fisher exact tests were used for two-way tables (The three treatment groups versus another nominal factor). When more than 30% of table cells had expected counts <5, then the Fisher exact test was calculated. For recovery time and interval to conception, values are expressed as the mean ± standard error. In all tests, a p≤0.05 was set as the significance level and the confidence interval was set at 95%.

## Results

The descriptive statistics of cows with luteal and follicular cysts and their distribution to treatment groups are shown in Table-1.

**Table-1:** The descriptive statistics of cows with luteal and follicular cysts and their distribution to treatment groups.

Cyst type	Treatment group			Total

	1	2	3	
Luteal	14	18	14	46
Follicular	33	29	36	98
Total	47	47	50	144

The recovery time, interval to conception, conception rate at first AI, and pregnancy rates by days 70 and 100 after treatment in cows with luteal or follicular cysts are shown in Table-2.

**Table-2:** Response to treatment in cows with luteal or follicular cysts.

Parameters	Treatment group				p value

	1	2	3	Total	
Recovery time	17±1.2	16.1±1	14.9±1.2	15.9±0.7	0.35
Interval to conception	57±7	66.4±9.2	53.4±6.2	58.8±4.3	0.81
Conception rate at first AI, % (n/n)	40.4 (19/47)	42.5 (20/47)	46 (23/50)	43.1 (62/144)	0.85
Pregnant by 70 days after treatment, % (n/n)	76.6 (36/47)	66 (31/47)	74 (37/50)	72.2 (104/144)	0.48
Pregnant by 100 days after treatment, % (n/n)	80.9 (38/47)	78.7 (37/47)	78 (39/50)	79.2 (114/144)	0.94

AI=Artificial insemination

There were no significant differences among groups in the recovery time, interval to conception, conception rate at first AI, and pregnancy rates by days 70 and 100 after treatment (p>0.05).

The recovery time, interval to conception, conception rate at first AI, and pregnancy rates by days 70 and 100 after treatment in cows with luteal cysts and follicular cysts are presented in Tables-3 and 4, respectively.

**Table-3:** Response to treatment in cows with luteal cysts.

Parameters	Treatment group				p value

	1	2	3	4	
Recovery time	14.3±1.7	15±1.4	13.4±2.1	14.3±1	0.4
Interval to conception	59.7±13.8	50.7±10.3	44.2±11.3	51.5±6.7	0.67
Conception rate at first AI, % (n/n)	28.6 (4/14)	55.6 (10/18)	50 (7/14)	45.6 (21/46)	0.29
Pregnant by 70 days after treatment, % (n/n)	71.4 (10/14)	61.1 (11/18)	85.7 (12/14)	71.7 (33/46)	0.33^a^
Pregnant by 100 days after treatment, % (n/n)	78.6 (11/14)	83.3 (15/18)	85.7 (12/14)	82.6 (38/46)	0.99^a^

a Fisher’s exact test was used. AI=Artificial insemination

**Table-4:** Response to treatment in cows with follicular cysts.

Parameters	Treatment group				p value

	1	2	3	4	
Recovery time	18.1±1.5	16.7±1.5	15.4±1.4	16.7±0.8	0.47
Interval to conception	55.9±8.2	76.1±13.3	57±7.3	62.3±5.5	0.61
Conception rate at first AI, % (n/n)	45.4 (15/33)	34.5 (10/29)	44.4 (16/36)	41.8 (41/98)	0.63
Pregnant by 70 days after treatment, % (n/n)	78.8 (26/33)	69 (20/29)	69.4 (25/36)	72.4 (71/98)	0.6
Pregnant by 100 days after treatment, % (n/n)	81.8 (27/33)	75.9 (22/29)	75 (27/36)	77.6 (76/98)	0.77

AI=Artificial insemination

No difference (p>0.05) was also found in all reproductive indices examined among groups of cows with luteal or follicular cysts in the present study.

## Discussion

According to the results of the present study, the recovery time, interval to conception, conception rate at first AI and pregnancy rates by days 70 and 100 after treatment were not different between GnRH/hCG plus cloprostenol groups and GnRH alone group. In contrast to our results, it was reported that simultaneous treatment of ovarian cysts with GnRH and cloprostenol resulted in better response to treatment compared to treatment with GnRH alone at the time of cyst diagnosis [27]. The difference between our findings and the above mentioned article could be due to the fact they used cows with a DIM beyond 57 days postpartum compared to the mean DIM of 48 (35-120 days) days postpartum in our study. It was reported that approximately 50% of cows developing ovarian cysts within 45 days of calving recovered spontaneously before 60 days postpartum [28]. In the present study, 36.1% (n=52) of ovarian cysts diagnoses were made before 40 days postpartum consisted of 40.4% (n=19) of cows in Group 1, 29.8% (n=14) of cows in Group 2, and 38% (n=19) of cows in Group 3, which was not significantly different between groups (p=0.53). Therefore, the rate of spontaneous recovery does not seem to be different between groups. Unfortunately, it was not practicable not to treat a group of cows with ovarian cysts to serve as control group because of farm limitations, and therefore we were not able to determine spontaneous recovery rate of cystic conditions. Another explanation for similar response to treatment of groups in this study might be the predominance of follicular cysts in three groups (70%, 62% and 72% in Groups 1, 2, and 3, respectively) which could have resulted in poor response to first PGF_2α_ treatment in Groups 2 and 3 and subsequently a response similar to that observed in Group 1. A poor response to PGF_2α_ treatment has previously been reported in cows with follicular cysts [21].

There were no significant differences among groups in the recovery time and interval to conception in cows with luteal cysts in the present study. Similarly, it was reported that when cows with ovarian cysts were simultaneously treated with GnRH and cloprostenol, there were no significant differences between groups in treatment to estrus interval or treatment to conception interval compared to those treated with GnRH alone [26]. We expected to observe a more effective response to simultaneous treatment with hCG or GnRH and cloprostenol than GnRH treatment alone in cows with luteal cyst in the present study. The pretreatment mean serum concentration of progesterone and in fact the presence of any active luteal tissue is a significant factor influencing the response to GnRH treatment [29,30]. On the other hand, it has been shown that the presence of inadequate luteal tissue in luteal cysts may elicit a poor response to the luteolytic effect of cloprostenol at the time of ovarian cyst diagnosis [21,31].

According to our results, the response of luteal cysts to the conventional treatment is not significantly different from that of the follicular cysts. Our reports are similar to Probo *et al*. [7] and in contrast to Sprecher *et al*. [16]. The similar response to GnRH treatment between luteal and follicular cysts could be explained by the fact that follicular cysts may luteinize in response to GnRH treatment and cows with luteal cysts may not respond to GnRH treatment, and then cows with either luteal or follicular cysts will subsequently respond to PGF_2α_ administered 10 days after GnRH treatment and this will result in a similar response to treatment.

## Conclusion

The results of the present study show that simultaneous treatment of ovarian cysts with hCG or GnRH and cloprostenol appeared to have no advantage over the conventional treatment, GnRH alone, in dairy cows. Furthermore, hCG and GnRH have an equal therapeutic effect in the treatment of bovine ovarian cysts. Therefore, the preferred treatment of ovarian cysts would be GnRH followed by PGF_2α_ 10 days later.

## Authors’ Contributions

MK designed the plan of the present study. TT and MH carried out the experimental work. Manuscript preparation along with data analysis was done by AM. MK and AM reviewed and revised the manuscript. All authors read and approved the final manuscript.

## References

[1] Vanholder T, Opsomer G, de Kruif A (2006). Aetiology and pathogenesis of cystic ovarian follicles in dairy cattle: A review. Reprod. Nutr. Dev.

[2] Garverick H.A (1997). Ovarian follicular cysts in dairy cows. J. Dairy Sci.

[3] Fourichon C, Seegers H, Malher X (2000). Effect of disease on reproduction in the dairy cow: A meta-analysis. Theriogenology.

[4] Lopez-Diaz M.C, Bosu W.T.K (1992). A review and an update of cystic ovarian degeneration in ruminants. Theriogenology.

[5] Borsberry S, Dobson H (1989). Periparturient diseases and their effect on reproductive performance in five dairy herds. Vet. Rec.

[6] Hooijer G.A, Lubbers R.B, Ducro B.J, van Arendonk J.A, Kaal-Lansbergen L.M, van der Lende T (2001). Genetic parameters for cystic ovarian disease in dutch black and white dairy cattle. J. Dairy Sci.

[7] Probo M, Comin A, Mollo A, Cairoli F, Stradaioli G, Veronesi M.C (2011). Reproductive performance of dairy cows with luteal or follicular ovarian cysts after treatment with buserelin. Anim. Reprod. Sci.

[8] Cattaneo L, Signorini M, Bertoli J, Bartolomé J, Gareis N, Díaz P, Bó G, Ortega H (2014). Epidemiological description of cystic ovarian disease in argentine dairy herds: Risk factors and effects on the reproductive performance of lactating cows. Reprod. Domest. Anim.

[9] Amweg A.N, Salvetti N.R, Stangaferro M.L, Paredes A.H, Lara H.H, Rodríguez F.M, Ortega H.H (2013). Ovarian localization of 11β-hydroxysteroid dehydrogenase (11βHSD):effects of ACTH stimulation and its relationship with bovine cystic ovarian disease. Domest. Anim. Endocrinol.

[10] Kahn C.M, Line S (2010). Cystic ovary disease. The Merck Veterinary Manual.

[11] Amiridis G.S (2009). Comparison of aspiration and hormonal therapy for the treatment of ovarian cysts in cows. Acta Vet. Hung.

[12] Roth Z, Biran D, Lavon Y, Dafni I, Yakobi S, Braw-Tal R (2012). Endocrine milieu and developmental dynamics of ovarian cysts and persistent follicles in postpartum dairy cows. J. Dairy Sci.

[13] Gundling N, Drews S, Hoedemaker M (2009). Comparison of two different programmes of ovulation synchronization in the treatment of ovarian cysts in dairy cows. Reprod. Domest. Anim.

[14] Rizzo A, Campanile D, Mutinati M, Minoia G, Spedicato M, Sciorsci R.L (2011). Epidural vs intramuscular administration of lecirelin, a GnRH analogue, for the resolution of follicular cysts in dairy cows. Anim. Reprod. Sci.

[15] Kawate N, Watanabe K, Uenaka K, Takahashi M, Inaba T, Tamada H (2011). Comparison of plasma concentrations of estradiol-17βand progesterone, and conception in dairy cows with cystic ovarian diseases between Ovsynch and Ovsynch plus CIDR timed AI protocols. J. Reprod. Dev.

[16] Sprecher D.J, Strelow L.W, Nebel R.L (1990). The response of cows with cystic ovarian degeneration to luteotropic or luteolytic therapy as assigned by latex agglutination milk progesterone assay. Theriogenology.

[17] el-Tahawy Ael-G, Fahmy M.M (2011). Partial budgeting assessment of the treatment of pyometra, follicular cysts and ovarian inactivity causing postpartum anoestrus in dairy cattle. Res. Vet. Sci.

[18] Rizzo A, Spedicato M, Mutinati M, Minoia G, Pantaleo M, Sciorsci R.L (2011). *In vivo* and *in vitro* studies of the role of the adrenergic system and follicular wall contractility in the pathogenesis and resolution of bovine follicular cysts. Theriogenology.

[19] Nakao T, Tomita M, Kanbayashi H, Takagi H, Abe T, Takeuchi Y, Ochiai H, Moriyoshi M, Kawata K (1992). Comparisons of several dosages of a GnRH analog with the standard dose of hCG in the treatment of follicular cysts in dairy cows. Theriogenology.

[20] De Rensis F, López-Gatius F, García-Ispierto I, Techakumpu M (2010). Clinical use of human chorionic gonadotropin in dairy cows:an update. Theriogenology.

[21] Leslie K.E, Bosu W.T (1983). Plasma progesterone concentrations in dairy cows with cystic ovaries and clinical responses following treatment with fenprostalene. Can. Vet. J.

[22] Chavatte P.M, Archbald L.F, Risco C, Tran T, Sumrall D (1993). Effectiveness of prostaglandin F2alpha in the initial treatment of bovine ovarian cysts. Theriogenology.

[23] Douthwaite R, Dobson H (2000). Comparison of different methods of diagnosis of cystic ovarian disease in cattle and an assessment of its treatment with a progesterone-releasing intravaginal device. Vet. Rec.

[24] Farin P.W, Youngquist R.S, Parfet J.R, Garverick H.A (1992). Diagnosis of luteal and follicular ovarian cysts by palpation per rectum and linear-array ultrasonography in dairy cows. J. Am. Vet. Med. Assoc.

[25] White M.E, Reimers T.J (1986). Milk progesterone concentrations following simultaneous administration of buserelin and cloprostenol in cattle with normal corpora lutea. Can. J Vet Res.

[26] Dinsmore R.P, White M.E, English P.B (1990). An evaluation of simultaneous GnRH and cloprostenol treatment of dairy cattle with cystic ovaries. Can. Vet. J.

[27] López-Gatius F, López-Béjar M (2002). Reproductive performance of dairy cows with ovarian cysts after different GnRH and cloprostenol treatments. Theriogenology.

[28] Morrow D.A, Roberts S.J, McEntee K (1969). Postpartum ovarian activity and involution of the uterus and cervix in dairy cattle. 1. Ovarian activity. Cornell. Vet.

[29] Dinsmore R.P, White M.E, Guard C.L, Jasko D.J, Perdrizet J.A, Powers P.M, Smith M.C (1989). Effect of gonadotropin-releasing hormone on clinical response and fertility in cows with cystic ovaries, as related to milk progesterone concentration and days after parturition. J. Am. Vet. Med. Assoc.

[30] Koppinen J, Vesanen M, Alanko M (1984). Ovarian cysts in dairy cattle-some aspects of diagnosis, treatment with GnRH and HCG and subsequent milk progesterone values. Nord. Vet. Med.

[31] Kesler D.J, Garverick H.A, Caudle A.B, Bierschwal C.J, Elmore R.G, Youngquist R.S (1978). Clinical and endocrine response of dairy cows with ovarian cysts to GnRH and PGF2α. J. Anim. Sci.

